# Fall prevention modulates decisional saccadic behavior in aging

**DOI:** 10.3389/fnagi.2012.00018

**Published:** 2012-07-12

**Authors:** Olivier A. Coubard

**Affiliations:** The Neuropsychological Laboratory, CNS-FedParis, France

**Keywords:** aging, motor control, eye movements, motor activity, plasticity

## Abstract

As society ages and frequency of falls increases in older adults, counteracting motor decline is a challenging issue for developed countries. Physical activity based on aerobic and strength training as well as motor activity based on skill learning both help benefit balance and reduce the risk of falls, as assessed by clinical or laboratory measures. However, how such programs influence motor control is a neglected issue. This study examined the effects of fall prevention (FP) training on saccadic control in older adults. Saccades were recorded in 12 participants aged 64–91 years before and after 2.5 months training in FP. Traditional analysis of saccade timing and dynamics was performed together with a quantitative analysis using the LATER model, enabling us to examine the underlying motor control processes. Results indicated that FP reduced the rate of anticipatory and express saccades in inappropriate directions and enhanced that of express saccades in the appropriate direction, resulting in decreased latency and higher left-right symmetry of motor responses. FP reduced within-participant variability of saccade duration, amplitude, and peak velocity. LATER analysis suggested that FP modulates decisional thresholds, extending our knowledge of motor training influence on central motor control. We introduce the Threshold Interval Modulation with Early Release-Rate of rIse Deviation with Early Release (TIMER-RIDER) model to account for the results.

## Introduction

In the last two decades, there has been an increasing interest in the aging of balance and its related disorders. Balance is a complex function that is achieved by (1) the integration of multiple sensory inputs such as visual, vestibular, and somesthetic afferences (2) central motor processing such as oculomotor and postural commands, and (3) generation of an adaptive response relying on muscle strength and power (Nashner, 1976). Aging induces changes in every component of balance: visual (Lord, 2006), vestibular (Kristinsdottir et al., 2001), proprioceptive and exteroceptive inputs (Famula et al., 2008), central processing (Horak, 2006), and muscle effectors (Schultz, 1995). Balance-related impairments have been described in aging such as reduced multisensory integration (Horak et al., 1989), increased visual dependency (Woollacott, 1993), increased motor tone (Judge, 2003), reduced speed of processing (Vance, 2009), reduced saccade latency (Shafiq-Antonacci et al., 1999), inversion of muscular activation sequences (Woollacott et al., 1986), all resulting in decreased balance and unsteady gait (Perrin et al., 1997). A meta-analysis reported that balance and gait disorders were the second risk factor for falls in older adults with dramatic related-injury and mortality consequences (Rubenstein, 2006). In this context, developing strategies to improve balance is a public health priority to preserve daily living independence and successful aging (Hank, 2011).

Several programs have been proposed to improve balance and reduce falls in aging, such as exercise, environmental inspection and hazard management, and interdisciplinary approaches have provided the best results (Rubenstein, 2006). Among exercise programs, home- or center-based fall prevention (FP) interventions have targeted strength and/or balance (e.g., Province et al., 1995; Campbell et al., 1999; Robertson et al., 2001), joint reinforcement, walking and stair climbing with weights, functional balance (King et al., 2002) or flexed posture (Benedetti et al., 2008). The efficacy of these programs has been assessed using either batteries such as the “Performance Oriented Mobility Assessment” (Tinetti, 1986), “Short Physical Performance” (Guralnik et al., 1994), “Frailty and Injuries/Cooperative Studies of Intervention Studies” (Rossiter-Fornoff et al., 1995), or clinical measures such as functional reach (Duncan et al., 1990) and the Berg Balance Scale (Berg et al., 1995), or laboratory measures such as stereophotogrammetric analysis (Benedetti et al., 2008). Tai Chi and dance have also been suggested as promising programs to boost balance and prevent falls in older adults (Judge, 2003). Nevertheless, there is a need for research to provide insight into motor control and help researchers understand how central processing improves after FP programs.

The purpose of this study was to examine the effects of FP on motor control in older fallers. To achieve this goal, their saccadic eye movements were measured before and after ~2.5 months' FP training. Not only was traditional analysis of timing and dynamics performed but also dynamic analysis of latency distributions. Saccades are interesting stereotyped movements since their underlying neural circuitry forms a well-defined system amenable to quantitative analysis that is likely to provide insightful information about central motor control (Carpenter, 2004). Saccade latency, the time interval elapsing between target onset and movement initiation, has been subjected to more studies than any other parameters. Traditionally, saccade latencies have been clustered into express (80–134 ms), fast regular (135–179 ms), slow regular (180–399 ms) and late (>400 ms) saccades (Fischer et al., 1997). Manipulating timing conditions between fixation point offset and target onset, numerous researches have explored whether the population of express saccades (80–120 or 80–134 ms in humans depending on authors) compared to the main distribution of saccades (>120 or >134 ms) may be due to either preparatory processes (e.g., Paré and Munoz, 1996) or the release of inhibitory processes enabling the retino-collicular route to bypass high-level decisional mechanisms (e.g., Isa and Kobayashi, 2004).

**Figure 1:**
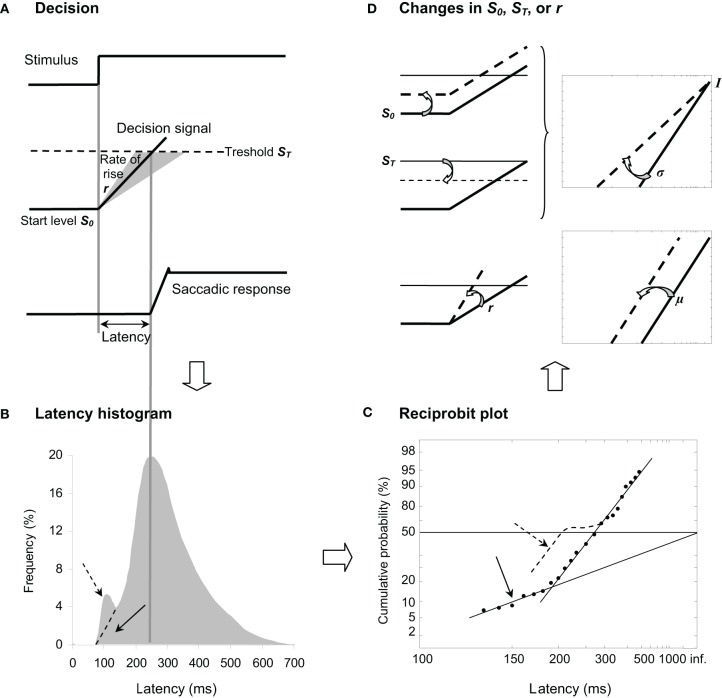
**(A)** LATER model. From *top to bottom*, we show the stimulus signal, the theoretical decision signal in a LATER unit, and the saccade signal. In response to the Stimulus, the Decision signal initiates at the Start level *S*_0_ and increases at a Rate of rise *r* until it reaches the Threshold *S*_*T*_ at which the Saccadic response is initiated. **(B)** Traditional histogram of Frequency (in %) as a function of Latency (in ms). The main distribution is skewed with a tail to the right, and some population of express (dashed arrow) vs. early (full arrow) saccades can occur exhibiting or not a distinct peak, respectively. **(C)** Resulting reciprobit plot of Cumulative probability on a probit scale (in %) as a function of Latency on a reciprocal scale (in ms). The main recinormal distribution lies on a straight line described by μ and σ. Early saccades can occur lying a second line described by σ′ (full arrow), while express saccades (dashed arrow) might exhibit a specific pattern (see Text). **(D)** Changes that can occur in LATER. A change in the distance between *S*_0_ and *S*_*T*_ results in a swivel of the recinormal distribution around the intercept (up). A change in the rate of rise *r* results in a lateral shift of the recinormal distribution (down).

Carpenter (1981) has highlighted that latency is surprisingly (1) long and (2) variable from trial-to-trial in contrast to what one might expect from the retino-collicular route, suggesting that a greater part of latency reflects decisional mechanisms resulting in a later response or some procrastination. In his LATER model (standing for Linear Approach to Threshold with Ergodic Rate), Carpenter (1999) suggested that, in response to a visual stimulus, some kind of decision signal starting at an initial level *S*_0_ rises at a constant rate *r* until it reaches a threshold value *S*_*T*_, at which point a response is initiated (see Figure 1A). Since latency and rate are reciprocally related, the author (Carpenter, 1981) proposed to examine not the distribution of latency T (see Figure 1B) but the distribution of its reciprocal 1/T (i.e., promptness) in a *reciprobit* plot defined as having a reciprocal scale in the *x* axis and a probit scale in the *y* axis (see Figure 1C), hence providing direct information about underlying decisional mechanisms. Indeed, if *r* randomly varies from trial-to-trial as a Gaussian with mean μ and variance σ^2^, the resulting *recinormal* distribution results in a straight line that is parsimoniously described by these two parameters. In some cases, a population of *early* latencies occur, lying on a second line whose intercept is 0.5 (see Figure 1C) (Carpenter, 1999). Recently, Noorani and Carpenter (2011) suggested that early saccades are different from express ones, which show a distinct peak in latency frequency histogram, and that they may exhibit a specific pattern in a reciprobit plot (see Figures 1B,C).

Since Carpenter's seminal research (Carpenter, 1981), thirty years of psychophysical experiments (e.g., Carpenter and Williams, 1995; Reddi and Carpenter, 2000) and of neurophysiological evidence (e.g., Hanes and Schall, 1996; Kim and Shadlen, 1999) have reinforced the view that LATER describes a genuine neurophysiological phenomenon. Two changes can occur in the recinormal distribution (see Figure 1D). First, the recinormal distribution can swivel around the intercept in the sense that latency is reduced (lower slope) or enhanced (higher slope) (see Figure 1D, up). Such a swiveling indicates a change in the distance between *S*_0_ and *S*_*T*_, and has been observed by manipulating either prior probability of target appearance thus altering *S*_0_ (Carpenter and Williams, 1995) or urgency given in the instruction to respond, thus altering *S*_*T*_ (Reddi and Carpenter, 2000). Second, the recinormal distribution can shift laterally in that latency is reduced (leftward shift) or enhanced (rightward shift), indicating a higher or lower supply of information as shown by a higher or lower rate of rise *r*, respectively (see Figure 1D, down). Such a change in *r* has enabled researchers to assess electrical stimulation of the subthalamic nucleus in Parkinson's disease (Temel et al., 2008).

Using LATER in addition to analysis of saccade timing and dynamics, the present study sought to examine the effects of FP on the underlying decisional mechanisms of saccade initiation, thus providing further insight into how FP influences motor control. Based on prior cross-sectional studies showing that older fallers exhibit fewer express saccades than non-fallers (Yang et al., 2008) and that older sporting adults have lower latency and higher accuracy of saccades than non-sporting adults (Gauchard et al., 2003), FP was expected to improve saccadic behavior in older adults. Specifically, we hypothesized that traditional saccade analysis would show higher accuracy and lower latency after training, possibly through an increase in the rate of express saccades. LATER analysis was expected to provide insight about whether such latency decrease is obtained through modulation by FP training of either decision thresholds *S*_0_ and/or *S*_*T*_ or decision gain *r*.

## Methods

### Participants

Twelve French elderly women gave their informed consent to participate in the study, which adhered to the tenets of the Declaration of Helsinki and was approved by the local ethics committee (Ecole des Hautes Etudes en Sciences Sociales, Paris). They were right-handed, had normal or corrected-to-normal vision, and were all unaware of the purpose of experiment.

Participants were aged 78.4 (Mean) ± 6.5 (SD) years (range = 64.7–91.1 years), had 11.0 ± 3.4 years of education, and scored 3.2 ± 0.7 to a 1–4 socio-cultural scale based on the level of education weighted by socio-professional experience (Kalafat et al., 2003). They scored 27.4 ± 2.0 out of 30 to the Mini-Mental State Examination assessing general mental abilities (Folstein et al., 1975), and 10.8 ± 1.6 out of 20 to the WAIS code assessing speed of processing (Wechsler, 1989).

Participants were recruited and examined in two geriatric hospitals. They all had a history of falling with at least one fall in the last year. They were given a medical prescription based on falling history to perform FP.

In addition to falling history, participant n°9 and n°10 suffered from depression for which they were treated with paroxetine and lithium, respectively. Participant n°11 had experienced and recovered from a stroke 14 months before the first examination for which she was treated with gabapentine.

### Stimuli and eye movement recording

Target presentation and measurement of saccades were performed by a miniaturised head-mounted saccadometer (Ober et al., 2003). This comprised three low-power lasers projecting high-contrast red targets in a horizontal line at −10°, 0°, and +10° in front of the participant, adjoined to a binocular 1 kHz infra-red scleral oculomoter, low-pass filtered at 250 Hz with 12 bit resolution. The system measured linearly within 5% horizontal eye movements up to ±35°. The spatial resolution was 0.1°.

### Procedure

Participants underwent saccadic recordings one week before and after the training intervention. In a dark room, participants sat facing a wall onto which targets were projected at a viewing distance of 2.5 m. Head movements were minimized with a chin rest. They were instructed to follow the red laser dots with their eyes as accurately and rapidly as they could. Every participant performed 100 saccades to targets chosen at random in left and right directions (resulting in 100.6 ± 7.1 and 99.4 ± 7.1 for left and right targets, respectively, over both periods), preceded by 32 calibration trials, 16 in each direction. When necessary, participants wore their usual spectacle correction.

A step or zero-gap paradigm was used. After a random foreperiod of 0.5–1 s, the central target (0°) was extinguished and simultaneously one of the angled targets (±10°) was turned on. After the saccade was detected, the target returned to the center to inaugurate the next trial. The overall duration of a recording consisting in 232 trials was ~7 min.

### Fall prevention program

Participants were trained to FP for ten weeks. Participants did not have a choice in the training in which they were enrolled, which was based on a medical prescription to prevent future falls. The training was conducted by two professional instructors (one in each hospital) and supervised by a senior teacher in the FP specialty. The frequency of the training was twice a week, and each session lasted 1 h and 15 min. Participants did not take part in any other motor programs during the intervention period.

FP focused on the development of balance and of lower limb strength. A session was organized in 4 stages. The first stage (15 min) was devoted to warm-up and stretching. The second stage (25 min) was dedicated to visual, vestibular, kinaesthetic, and proprioceptive exercices. In visual exercices, participants had to increase their visual scanning frequency and amplitude while walking, and to step over obstacles with their eyes open or with their eyes closed. In vestibular exercices, they had to saccade leftward or rightward with their head while walking. In kinaesthetic exercices, they had to mimic movements of hands, arms, trunk, legs, and feet made by the trainer, such as pointing, raising or stretching one arm, at different paces. In proprioceptive exercices, they had to massage their feet using a ball or bag of sand. The third stage (25 min) involved workshops around objects: participants had to step over obstacles, to walk on foams, rubbers, and small bags of sand, to walk on a rope, etc. The training focused on the reinforcement of lower limb strength and on the improvement of balance to ensure postural stability, as well as accuracy and amplitude of movements. Finally, the fourth stage (10 min) consisted in cooling-down and stretching. The training was performed individually and in pairs.

### Saccadic measurements

Following acquisition, the data was downloaded onto a computer running LatencyMeter 4.9 (Ober et al., 2003), which automatically performed calibration. Saccades contaminated by blinks or of abnormal profile were rejected using an automatic procedure that examined the whole ensemble of traces and eliminated all those for which the position or velocity profile fell outside the normal range. This examination was based on log likelihood value for each sample of a given trace according to the mean and standard deviation calculated from the whole population of traces for that sample. The trace was rejected if the average log likelihood value for whole trace was below the rejection threshold. Other criteria for rejection were saccade detection failure and sensor range saturation. In all, 13.6% of trials (range = 7.0–26.0%) were discarded from the analysis.

Using home-made scripts under Matlab 7.0 (The MathWorks, US), we calculated the rate of anticipatory saccades (0–80 ms), express saccades (80–134 ms), fast regular saccades (135–179 ms), slow regular saccades (180–399 ms), late saccades (400–699 ms), very late saccades (700–999 ms) and saccades above the upper limit (1 s) in valid (left and right saccades in response to left and right targets, respectively) and invalid (left and right saccades in response to right and left targets, respectively) directions. The limits for express, fast regular, slow regular, and late saccades were those recommended by Fischer et al. (Fischer et al., 1997). The upper limit was set to 1 s as participants were aged and some of them under medication.

Importantly, the term “express saccades” is used here for saccades having latency between 80 and 134 ms and *exhibiting or not* a distinct peak in frequency histograms (Fischer et al., 1997), and the term “early saccades” for the early population in reciprobit plots (Carpenter, 1999). It is out of the scope of the present study to discuss the express nature of short-latency saccades, keeping in mind that the examination of latency distribution is not a definite method to identify express saccades (Krauzlis and Miles, 1996), be it a frequency histogram or a reciprobit plot. However, in line with a recent report (Noorani and Carpenter, 2011), an attempt to disentangle the two types of short-latency saccades will be made in the Discussion section.

For only valid direction, we built traditional distributions of latencies including anticipatory responses for every participant and for the group using a frequency histogram. For only visually guided saccades (i.e., with latency between 80 ms and 1 s) in only valid direction, we calculated the mean latency, duration, amplitude, gain (saccade amplitude divided by target eccentricity) and peak velocity. The within-participant standard deviation or variability was also calculated for latency, duration, amplitude, and peak velocity of those visually guided saccades in the valid direction.

Using SPIC 15.iv.2010 (Carpenter, 1994), we built a cumulative reciprobit frequency histogram of latency of visually guided saccades (80–1000 ms) in valid direction and fitted two trendlines to the distribution: the first passing through the population for standard saccades, the second through any population of early saccades (Carpenter, 1999). We calculated the mean μ and standard deviation σ of the main distribution of saccades and σ′ of any population of early saccades for every participant and for the group.

### Statistical analysis

Using Statistica 7.0 (StatSoft, US), analyses of variance (ANOVAs) with Target location (two levels: left or right) and Period (two levels: pre- or post-test period) as within-participant factors were performed on the rates of saccades in valid and invalid directions (anticipatory, express, fast regular, slow regular, late, very late saccades) and metrics of saccades in valid direction (mean and variability of latency, duration, amplitude, gain, peak velocity; μ, σ, and σ′). *Post-hoc* tests were calculated using the Least Significant Difference (LSD) test. Critical results were re-examined using the Wilcoxon test. At the individual level and for only valid direction, a χ^2^ test was done between pre- and post-test periods for the number of anticipatory and of express latencies.

Using SPIC 15.iv.2010 (Carpenter, 1994), recinormal distributions between the two periods or target locations were compared using the Kolmogorov-Smirnov (KS) two-sample test. As the means μ appeared Gaussian distributed as confirmed using the Anderson–Darling test, Student's *t* tests could appropriately be used to compare the two periods. Finally, we tested the swivel vs. shift of distributions between the two periods. To achieve this goal, data were fitted with each of the two constraints in turn using maximum likelihood and we reported which logarithm of the ratio of the two likelihoods was favored over the other. These statistics were performed for every participant and for the group.

## Results

### Populations of anticipatory and express saccades

**Figure 2:**
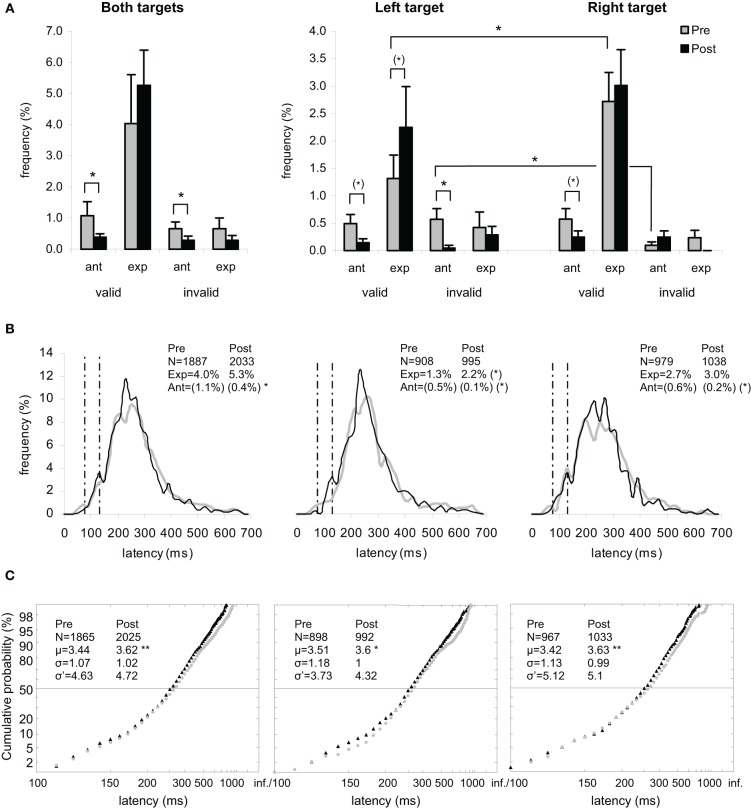
**Results for the group of participants in Pre-test (gray) and Post-test (black) periods for Both targets (left panels), Left target (middle panels), and Right target (right panels). (A)** Rates of anticipatory (0–80 ms) and of express (80–134 ms) saccades, separately for valid (i.e., correct responses) and invalid (i.e., errors) directions. **(B)** Histograms of Frequency (in %) as a function of Latency (in ms) for saccades between 0 and 999 ms in valid direction, and rates of express (Exp) and of anticipatory (Ant) saccades. Bin width is 20 ms. Vertical semi-dotted lines indicate the limits of express saccades: 80 and 134 ms. **(C)** Reciprobit plots of Cumulative probability (in %) as a function of Latency (in ms) for saccades between 80 and 999 ms in valid direction, and μ, σ, and σ′ values. **(A–C)** Asterisks indicate statistical significant differences (^**^P < 0.01, ^*^P < 0.05, (^*^).05 < *P* < 0.10). N indicates the number of trials.

**Figure 3:**
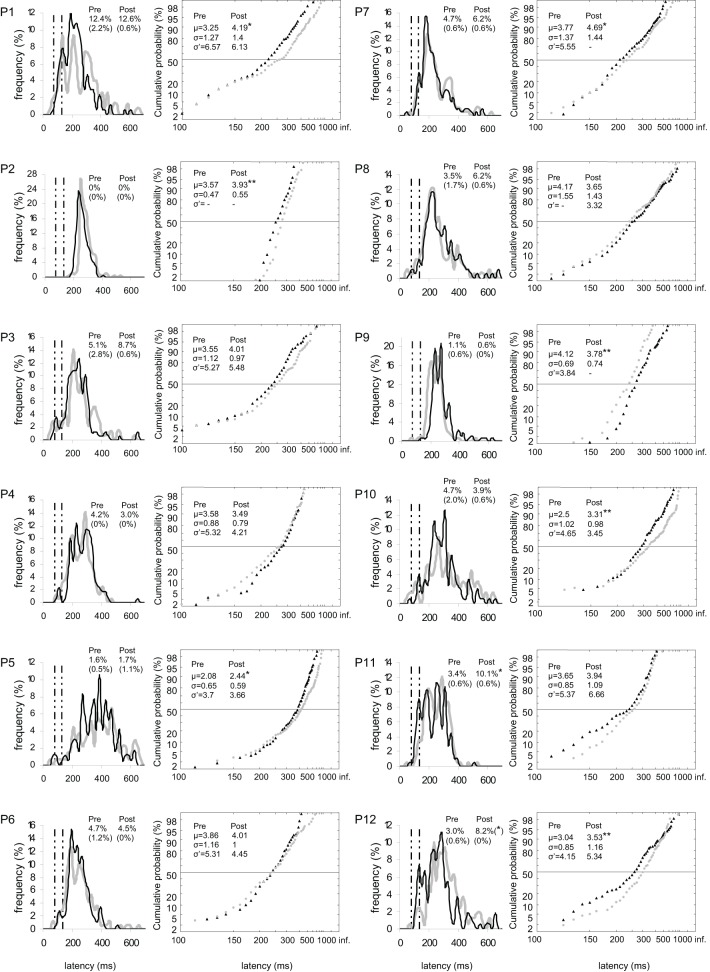
**Results for every participant in Pre-test (gray) and Post-test (black) periods for both targets.** (Columns 1, 3) Histograms of Frequency (in %) as a function of Latency (in ms) for saccades between 0 and 999 ms in valid direction, and rates of express (Exp) and of anticipatory (Ant) saccades. Bin width is 20 ms. Vertical semi-dotted lines indicate the limits of express saccades: 80 and 134 ms. (Columns 2, 4) Reciprobit plots of Cumulative probability (in %) as a function of Latency (in ms) for saccades between 80 and 999 ms in valid direction, and μ, σ, and σ′ values. Asterisks indicate statistical significant differences (^**^P < 0.01, ^*^P < 0.05, (^*^).05 < *P* < 0.10). *N* = 133–185 in frequency histograms; *N* = 130–185 in reciprobit plots.

Results are shown in Figures 2A,B for the group of participants and in Figures 3, 5 (columns 1 and 3) for, respectively both, left, and right targets in every participant.

#### Valid direction

For the rate of anticipatory saccades (0–80 ms), ANOVA with Target location (2 levels: left or right) and Period (2 levels: pre- or post-test period) as factors showed a main effect of Period [*F*_(1, 11)_ = 7.86, *P* = 0.017] with decreasing rates between pre- (1.07%) and post-test (0.39%) periods, which was confirmed by Wilcoxon test (*Z* = 2.39, *P* = 0.017) (see Figures 2A,B, left panel). Though the two factors did not interact (*F* < 1), *post-hoc* test showed a marginal Period effect with decreasing rates between pre- and post-test periods for left target (0.49 vs. 0.14%; LSD, *P* = 0.073; Wilcoxon, *Z* = 2.03, *P* = 0.042) and right target (0.58 vs. 0.25%; LSD, *P* = 0.088; Wilcoxon, *Z* = 1.54, *P* = 0.123), as shown in Figures 2A,B (middle and right panels).

For the rate of express saccades (80–134 ms), ANOVA with Target location and Period as factors showed a marginal effect of Target [*F*_(1, 11)_ = 3.97, *P* = 0.072] with lower value for left target (3.57%) than for right target (5.73%), which was confirmed by Wilcoxon test (*Z* = 2.04, *P* = 0.041). Though there was no interaction (*F* < 1), *post-hoc* test showed a marginal Period effect with increasing rates between pre- and post-test periods for left target (1.32 vs. 2.25%; LSD, *P* = 0.073; Wilcoxon, *Z* = 1.27, *P* = 0.203), which was not the case for right target (2.72 vs. 3.01%; LSD, *P* = 0.547). This pattern resulted in a left-right asymmetry in the pre-test period (1.32 vs. 2.72% for left and right targets, respectively; LSD, *P* = 0.012; Wilcoxon, *Z* = 2.80, *P* = 0.005), which was no longer present in the post-test period (2.25 vs. 3.01%; LSD, *P* = 0.132), as illustrated in Figures 2A,B (middle and right panels).

**Figure 4:**
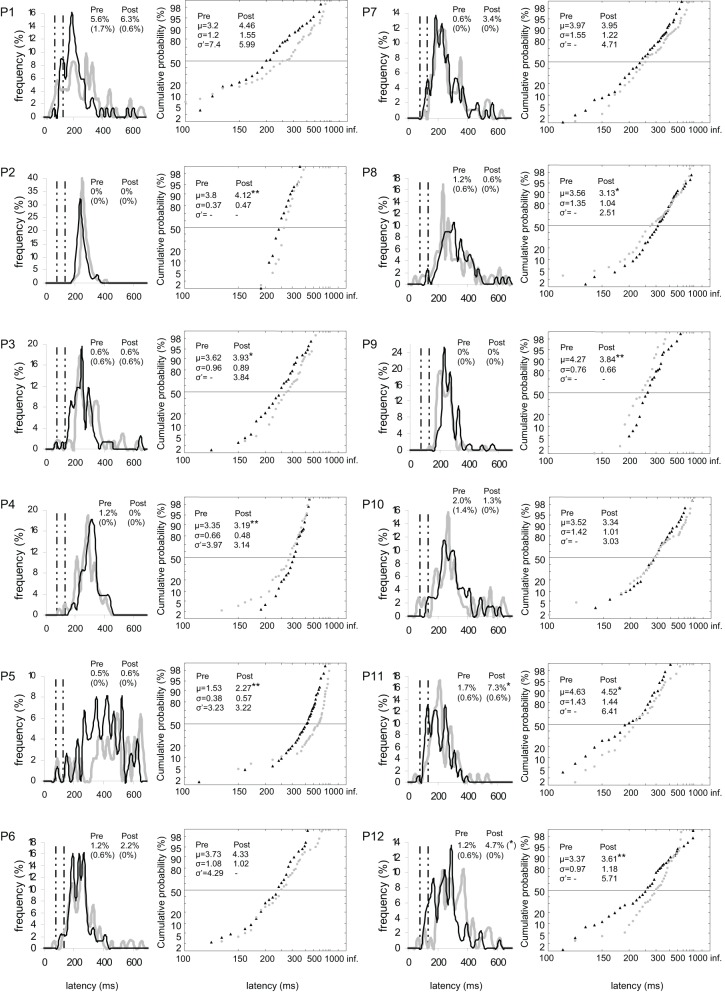
**Results for every participant in Pre-test (gray) and Post-test (black) periods for left target.** (Columns 1, 3) Histograms of Frequency (in %) as a function of Latency (in ms) for saccades between 0 and 999 ms in valid direction, and rates of express (Exp) and of anticipatory (Ant) saccades. (Columns 2, 4) Reciprobit plots of Cumulative probability (in %) as a function of Latency (in ms) for saccades between 80 and 999 ms in valid direction, and μ, σ, and σ′ values. *N* = 47–100 in frequency histograms and reciprobit plots. Other notations as in Figure 3.

**Figure 5:**
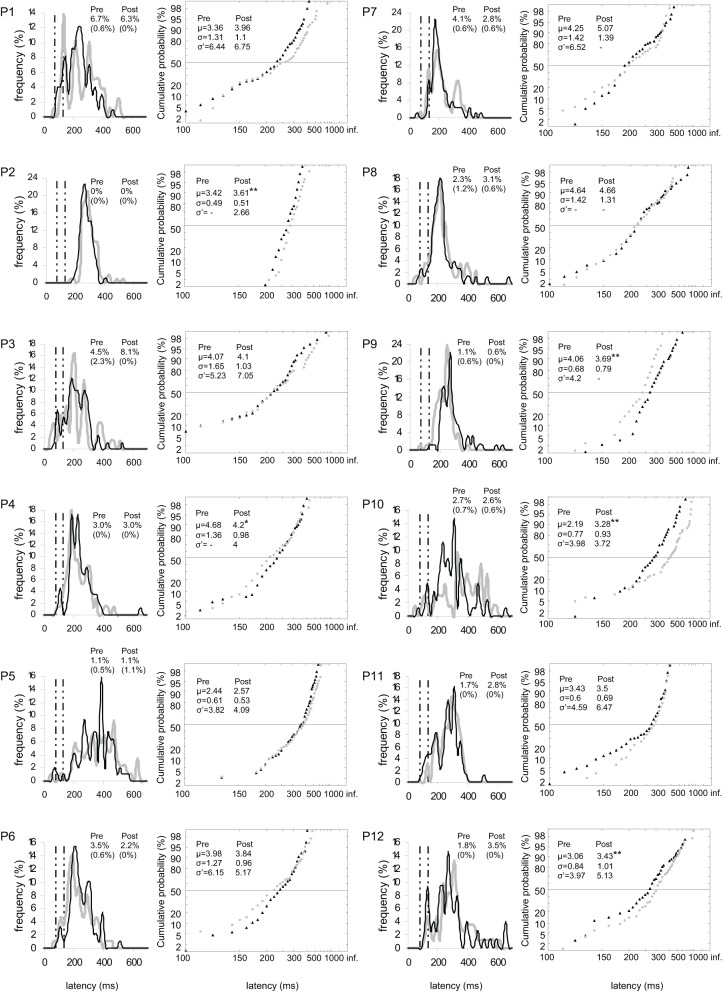
**Results for every participant in Pre-test (gray) and Post-test (black) periods for right target.** (Columns 1, 3) Histograms of Frequency (in %) as a function of Latency (in ms) for saccades between 0 and 999 ms in valid direction, and rates of express (Exp) and of anticipatory (Ant) saccades. (Columns 2, 4) Reciprobit plots of Cumulative probability (in %) as a function of Latency (in ms) for saccades between 80 and 999 ms in valid direction, and μ, σ, and σ′ values. *N* = 63–106 in frequency histograms; *N* = 62–106 in reciprobit plots. Other notations as in Figure 3.

At the individual level, the rate of anticipatory saccades tended to decrease between pre- and post-test periods in seven participants for both targets (n°1, 3, 6, 8–10, and 12; see Figure 3), and in five participants for left target (n°1, 6, 8, 10, and 12; see Figure 4) and right target (n°1, 3, 6, 8, and 9; see Figure 5). In contrast, the rate of express saccades showed an increasing trend between the two periods in five participants for both targets (n°3, 7, 8, 11, and 12; see Figure 3) and left target (n°1, 6, 7, 11, and 12; see Figure 4), and in four participants for right target (n°3, 8, 11, and 12; see Figure 5). For both targets and left target, this Period effect was, respectively, significant and marginally significant in participants n°11 and 12 (χ^2^ test; see Figures 3 and 5).

#### Invalid direction

For the rate of anticipatory saccades, ANOVA with Target location and Period as factors showed a marginal effect of Target [*F*_(1, 11)_ = 3.52, *P* = 0.087; Wilcoxon, *Z* = 1.52, *P* = 0.128] with a tendency for higher rate for left target (0.62%) than for right target (0.34%), and a main effect of Period [*F*_(1, 11)_ = 9.04, *P* = 0.012] with decreasing rates between pre- (0.66%) and post-test (0.29%) periods, which was confirmed by Wilcoxon test (*Z* = 2.37, *P* = 0.018) (see Figures 2A,B, left panel). The interaction between the two factors was significant [*F*_(1, 11)_ = 5.73, *P* = 0.036] as a result of left target eliciting lower rates between pre- (0.57%) and post-test (0.05%) periods (LSD, *P* = 0.023; Wilcoxon, *Z* = 2.37, *P* = 0.018), which was not the case of right target showing statistically similar rates in the two periods (0.10 vs. 0.24%; LSD, *P* = 0.465). In other words, the left-right asymmetry in pre-test period (0.57 vs. 0.10% for left and right targets, respectively; LSD, *P* = 0.036; Wilcoxon, *Z* = 2.20, *P* = 0.028) disappeared in post-test period with values getting close to ground (0.05 vs. 0.24% for left and right targets; LSD, *P* = 0.340), as illustrated in Figures 2A,B (middle and right panels).

For the rate of express saccades, there was insufficient number of participants making errors to perform statistics.

### Populations of regular saccades

For valid direction, the rates of fast regular saccades (135–179 ms), slow regular saccades (180–399 ms), late saccades (400–699 ms), and very late saccades (700–999 ms) were, respectively, 7.95 ± 5.58%, 66.1 ± 19.1%, 9.84 ± 9.33%, 2.23 ± 2.90% in the pre-test period, and 9.09 ± 6.91%, 73.8 ± 13.8%, 8.53 ± 10.3%, 0.94 ± 1.01% in the post-test period. ANOVAs with Target location and Period as factors showed neither main effects nor interaction for the rates of fast regular, late and very late saccades. For slow regular saccades, there was a main effect of Period [*F*_(1, 11)_ = 5.58, *P* = 0.038] with increasing rates between pre- (66.1%) and post-test (73.8%) periods.

For invalid direction, the rates of fast regular saccades, slow regular saccades, late saccades, and very late saccades were 0.48 ± 1.30%, 2.32 ± 2.56%, 1.68 ± 3.18%, 0.70 ± 1.36% in the pre-test period, and 0.05 ± 0.16%, 0.49 ± 0.71%, 0.24 ± 0.66%, 0.20 ± 0.39% in the post-test period. ANOVAs with Target location and Period as factors showed neither main effects nor interaction for the rates of late and very late saccades. For slow regular saccades, there was a main effect of Period [*F*_(1, 11)_ = 9.05, *P* = 0.012] with decreasing rates between pre- (2.32%) and post-test (0.49%) periods. For fast regular saccades, there were insufficient errors to perform statistics.

The rate of saccades above the upper limit (>1 s) was 0.68 ± 1.08% of trials (range = 0–3.49%) when pooling valid and invalid directions over both periods. These saccades were not subjected to further analysis.

### Saccade timing and dynamics

Results are detailed in Tables 1–4 for, respectively latency, duration, amplitude, and peak velocity for every participant and for the group.

**Table 1:** **Mean ± Standard deviation of latency in milliseconds for both, left, and right targets in the two periods, for every participant and the group of participants (All)**.

	**Both targets**		**Left target**		**Right target**	
	**Pre**	**Post**	**Pre**	**Post**	**Pre**	**Post**
P1	282.3 ± 151.1	230.9 ± 99.6	289.1 ± 160.6	230.3 ± 122.4	276.4 ± 142.1	231.3 ± 80.7
P2	287.2 ± 47.6	261.1 ± 38.9	269.0 ± 35.2	248.3 ± 34.3	299.7 ± 50.8	276.1 ± 38.6
P3	274.2 ± 128.0	244.0 ± 104.0	292.7 ± 93.2	259.6 ± 90.6	255.2 ± 153.7	230.9 ± 112.3
P4	261.3 ± 91.7	272.0 ± 75.8	288.4 ± 95.9	307.4 ± 57.3	227.8 ± 73.4	238.6 ± 76.0
P5	443.5 ± 185.5	382.0 ± 124.4	563.4 ± 210.7	415.8 ± 139.6	378.0 ± 129.5	354.7 ± 102.9
P6	255.9 ± 111.2	246.4 ± 84.2	273.1 ± 117.4	245.8 ± 95.0	239.5 ± 102.2	246.9 ± 72.4
P7	261.7 ± 121.7	236.6 ± 89.7	294.5 ± 139.5	253.7 ± 96.4	221.4 ± 78.5	216.4 ± 76.2
P8	279.1 ± 136.4	298.5 ± 153.8	326.4 ± 155.4	347.3 ± 156.2	239.7 ± 102.9	249.0 ± 134.2
P9	239.2 ± 49.0	279.2 ± 74.7	241.3 ± 50.1	272.1 ± 66.7	236.7 ± 47.5	285.7 ± 80.9
P10	381.1 ± 194.1	308.7 ± 124.4	338.8 ± 174.9	316.3 ± 139.1	427.5 ± 203.4	301.9 ± 109.2
P11	256.8 ± 79.1	233.5 ± 85.7	236.9 ± 92.9	212.3 ± 88.2	272.2 ± 62.3	255.6 ± 77.0
P12	323.0 ± 136.2	283.4 ± 147.9	313.8 ± 101.7	283.2 ± 165.4	331.4 ± 160.9	283.7 ± 124.1
All	295.4 ± 119.3	273.0 ± 100.2	310.6 ± 118.9	282.7 ± 104.3	283.8 ± 108.9	264.2 ± 90.4

**Table 2:** **Mean ± Standard deviation of duration in milliseconds for both, left, and right targets in the two periods, for every participant and the group of participants (All)**.

	**Both targets**		**Left target**		**Right target**	
	**Pre**	**Post**	**Pre**	**Post**	**Pre**	**Post**
P1	50.1 ± 13.6	54.1 ± 7.6	53.1 ± 16.1	53.6 ± 7.0	47.5 ± 10.2	54.5 ± 8.0
P2	57.7 ± 5.6	58.5 ± 5.3	54.8 ± 3.8	56.7 ± 4.4	59.7 ± 5.8	60.7 ± 5.5
P3	54.7 ± 17.6	51.2 ± 7.4	52.5 ± 4.8	54.2 ± 5.1	57.0 ± 24.4	48.6 ± 8.1
P4	52.0 ± 4.6	54.3 ± 4.4	50.8 ± 4.0	54.4 ± 4.3	53.5 ± 4.8	54.3 ± 4.5
P5	52.1 ± 8.9	55.1 ± 6.3	49.5 ± 8.7	54.6 ± 5.8	53.5 ± 8.6	55.5 ± 6.6
P6	51.9 ± 8.4	48.8 ± 6.9	54.3 ± 6.2	50.7 ± 5.1	49.6 ± 9.5	46.8 ± 7.8
P7	61.0 ± 11.1	52.0 ± 9.5	59.6 ± 12.0	55.2 ± 9.4	62.8 ± 9.5	48.3 ± 8.3
P8	54.3 ± 15.2	56.3 ± 6.8	55.2 ± 21.1	57.0 ± 5.8	53.6 ± 7.1	55.7 ± 7.6
P9	57.3 ± 13.4	54.1 ± 13.0	66.4 ± 11.2	63.9 ± 12.4	46.1 ± 4.4	45.1 ± 3.5
P10	46.4 ± 11.9	47.0 ± 7.6	46.2 ± 11.2	46.7 ± 8.2	46.7 ± 12.7	47.2 ± 7.0
P11	52.9 ± 8.3	50.3 ± 4.9	55.5 ± 10.2	50.2 ± 5.2	50.8 ± 5.5	50.3 ± 4.7
P12	56.2 ± 9.6	58.5 ± 7.1	51.5 ± 9.4	57.7 ± 6.1	60.5 ± 7.6	59.5 ± 8.0
All	53.9 ± 10.7	53.4 ± 7.2	54.1 ± 9.9	54.6 ± 6.6	53.4 ± 9.2	52.2 ± 6.6

**Table 3:** **Mean ± Standard deviation of amplitude in degrees for both, left, and right targets in the two periods, for every participant and the group of participants (All)**.

	**Both targets**		**Left target**		**Right target**	
	**Pre**	**Post**	**Pre**	**Post**	**Pre**	**Post**
P1	10.5 ± 4.4	10.1 ± 3.0	11.3 ± 5.0	10.0 ± 2.6	9.8 ± 3.7	10.1 ± 3.3
P2	9.8 ± 0.8	9.9 ± 0.6	9.8 ± 0.7	9.8 ± 0.6	9.8 ± 0.9	9.9 ± 0.6
P3	9.4 ± 2.3	9.6 ± 1.5	8.1 ± 0.9	9.4 ± 1.0	10.7 ± 2.6	9.7 ± 1.9
P4	9.8 ± 1.4	9.7 ± 1.0	9.6 ± 1.4	9.7 ± 0.9	10.0 ± 1.4	9.8 ± 1.0
P5	10.0 ± 2.9	9.8 ± 1.1	10.4 ± 3.8	9.9 ± 1.0	9.8 ± 2.3	9.8 ± 1.1
P6	10.0 ± 2.8	9.8 ± 1.0	10.0 ± 1.5	9.8 ± 0.8	10.0 ± 3.7	9.9 ± 1.1
P7	9.7 ± 1.6	9.7 ± 1.3	9.6 ± 1.6	9.7 ± 1.5	9.7 ± 1.5	9.6 ± 1.1
P8	10.1 ± 2.0	9.6 ± 1.7	10.0 ± 2.0	9.9 ± 1.4	10.3 ± 2.0	9.4 ± 1.9
P9	9.7 ± 1.0	9.8 ± 1.0	9.1 ± 0.7	9.2 ± 0.8	10.3 ± 0.9	10.4 ± 0.8
P10	9.6 ± 2.3	9.8 ± 1.5	9.2 ± 1.7	9.6 ± 1.2	10.0 ± 2.8	9.9 ± 1.6
P11	9.8 ± 1.2	9.7 ± 1.3	9.6 ± 1.2	9.8 ± 1.2	9.9 ± 1.2	9.7 ± 1.3
P12	9.5 ± 1.9	9.8 ± 1.7	9.1 ± 2.0	9.7 ± 1.6	9.8 ± 1.8	9.9 ± 1.8
All	9.8 ± 2.1	9.8 ± 1.4	9.7 ± 1.9	9.7 ± 1.2	10.0 ± 2.1	9.8 ± 1.5

**Table 4:** **Mean ± Standard deviation of peak velocity in degrees per second for both, left, and right targets in the two periods, for every participant and the group of participants (All)**.

	**Both targets**		**Left target**		**Right target**	
	**Pre**	**Post**	**Pre**	**Post**	**Pre**	**Post**
P1	405.8 ± 127.6	348.4 ± 74.9	436.4 ± 157.8	363.4 ± 70.2	379.1 ± 85.0	338.2 ± 76.3
P2	308.8 ± 26.9	314.2 ± 35.4	320.4 ± 25.3	342.1 ± 17.3	300.9 ± 25.0	281.4 ± 19.9
P3	400.6 ± 150.2	405.4 ± 105.7	308.3 ± 39.3	324.2 ± 46.1	495.9 ± 162.5	474.0 ± 92.4
P4	343.1 ± 39.5	319.0 ± 31.3	341.1 ± 42.5	304.8 ± 27.1	345.7 ± 35.3	332.4 ± 29.1
P5	353.3 ± 77.6	342.5 ± 26.7	384.4 ± 92.3	346.4 ± 26.7	336.3 ± 62.0	339.3 ± 26.4
P6	380.0 ± 88.7	451.6 ± 44.5	388.4 ± 58.5	434.4 ± 33.6	372.0 ± 109.4	468.0 ± 47.4
P7	331.4 ± 45.2	398.5 ± 64.2	339.9 ± 47.7	363.7 ± 51.2	320.9 ± 39.4	439.8 ± 52.5
P8	361.3 ± 57.2	333.6 ± 37.2	375.6 ± 56.9	343.0 ± 34.0	349.5 ± 54.7	324.1 ± 37.8
P9	425.5 ± 40.8	426.2 ± 38.3	402.5 ± 36.3	397.4 ± 26.9	454.0 ± 25.2	452.7 ± 26.2
P10	428.4 ± 97.6	429.3 ± 64.6	424.2 ± 88.2	432.8 ± 61.0	433.0 ± 106.7	426.2 ± 67.5
P11	385.4 ± 34.1	399.1 ± 36.2	375.0 ± 30.2	394.2 ± 36.6	393.4 ± 34.9	404.2 ± 35.2
P12	337.2 ± 57.4	321.9 ± 48.0	378.0 ± 49.9	325.0 ± 47.8	299.8 ± 33.4	318.2 ± 48.1
All	371.7 ± 70.2	374.1 ± 50.6	372.9 ± 60.4	364.3 ± 39.9	373.4 ± 64.5	383.2 ± 46.6

For mean latency, ANOVA with Target location and Period as factors showed a main effect of Period [*F*_(1, 11)_ = 5.08, *P* = 0.046; Wilcoxon, *Z* = 1.96, *P* = 0.049] with decreasing latency between pre- (295.4 ms) and post-test (273.0 ms) periods. For variability of latency, the Period effect was marginally significant [*F*_(1, 11)_ = 4.09, *P* = 0.068; Wilcoxon, *Z* = 1.80, *P* = 0.072] with a tendency for decreasing values between pre- (119.3 ms) and post-test (100.2 ms) periods.

For mean duration, amplitude, gain, and peak velocity, ANOVAs with Target location and Period as factors showed neither main effects nor interaction. In contrast, for variability, there was a main effect of Period for duration [*F*_(1, 11)_ = 12.9, *P* = 0.004; Wilcoxon, *Z* = 3.06, *P* = 0.002], amplitude [*F*_(1, 11)_ = 10.2, *P* = 0.009; Wilcoxon, *Z* = 2.90, *P* = 0.004], and peak velocity [*F*_(1, 11)_ = 9.50, *P* = 0.010; Wilcoxon, *Z* = 2.20, *P* = 0.028] with decreasing values between the two periods for all parameters (10.7 vs. 7.2 ms, 2.1 vs. 1.4°, 70.2 vs. 50.6°/s for, respectively duration, amplitude and peak velocity).

### Later analysis

Results are detailed in Table 5 and shown in Figure 2C for the group of participants and in Figures 3–5 (columns 2 and 4) for, respectively both, left and right targets in every participant.

**Table 5:** ***D*-values of Kolmogorov-Smirnov (KS), and Swivel vs. Shift (Swi/Shi) tests on recinormal latency distributions between pre- and post-periods for every participant and the group of participants (All)**.

	**Both targets**			**Left target**			**Right target**		
	**KS**	**Swi/Shi**	**N**	**KS**	**Swi/Shi**	**N**	**KS**	**Swi/Shi**	**N**
P1	0.255^**^	swi^**^	146–166	0.306^**^	swi^**^	68–67	0.219^*^	swi^**^	78–99
P2	0.330^**^	swi	179–185	0.393^**^	swi	73–100	0.212^*^	shi	106–85
P3	0.177^*^	swi	130–166	0.226	swi	66–76	0.136	swi^**^	64–90
P4	0.119	swi	163–169	0.205	swi^*^	90–82	0.142	swi^**^	73–87
P5	0.191^**^	shi	133–168	0.420^**^	swi	47–75	0.128	swi	86–93
P6	0.082	swi	166–178	0.146	shi	81–87	0.150	swi	85–91
P7	0.105	swi^**^	156–175	0.133	swi^**^	86–95	0.105	swi^**^	70–80
P8	0.088	swi^**^	154–155	0.167	swi^**^	70–78	0.074	swi^**^	84–77
P9	0.263^**^	shi	177–173	0.300^**^	swi	98–83	0.320^**^	shi	79–90
P10	0.217^**^	swi^**^	130–151	0.109	swi^**^	68–71	0.429^**^	swi^**^	62–80
P11	0.166^*^	swi^**^	172–176	0.169	swi^**^	75–90	0.129	swi^**^	97–86
P12	0.215^**^	swi^**^	159–163	0.237^*^	swi^**^	76–88	0.208	swi^**^	83–75
All	0.063^**^	swi^**^	1865–2025	0.058	swi^**^	898–992	0.080^**^	swi^**^	967–1033

Statistical significance: ^**^P < 0.01; ^*^P < 0.05. *N* is the number of saccades in pre- and post-test periods.

#### Main distributions

For μ, ANOVA with Target location and Period as factors showed a marginal Period effect [*F*_(1, 11)_ = 4.37, *P* = 0.060; Wilcoxon, *Z* = 2.35, *P* = 0.019] with an increasing trend between pre- (3.44) and post-test (3.62) periods, which was confirmed by a significant difference in Student's *t* test (*t* = −4.71, *P* = 0.001) (see Figure 2C, left panel). Though there was no interaction (*F* < 1), *post-hoc* test showed a significant and marginal Period effect with increasing values between the two periods for, respectively, left target (3.51 vs. 3.60; LSD, *P* = 0.009; Wilcoxon, *Z* = 1.96, *P* = 0.049; *t* = −3.24, *P* = 0.002) and right target (3.42 vs. 3.63; LSD, *P* = 0.090; Wilcoxon, *Z* = 1.49, *P* = 0.136; *t* = −3.45, *P* = 0.001), as shown in Figure 2C (middle and right panels).

For σ, ANOVA with Target location and Period as factors showed neither main effects nor interaction.

At the individual level, μ significantly increased in six participants for both targets (n°1, 2, 5, 7, 10, and 12; see Figure 3), in four participants for left target (n°2, 3, 5, and 12; see Figure 4), and in three participants for right target (n°2, 10, and 12; see Figure 5).

#### Early distributions

The occurrence of early saccades depended on participants and/or conditions (see Figures 3–5). When no early saccades occurred, σ′ was either given the value zero or no value. Retaining the zeros, three statistics were first performed as follows. First, ANOVA on σ′ with Target location and Period as factors showed neither main effects nor interaction. Second, Wilcoxon test between pre- and post-test periods failed to reach significance for both targets (4.63 vs. 4.72; *Z* = 1.24, *P* = 0.213), left target (3.73 vs. 4.32; *Z* < 1), and right target (5.12 vs. 5.10; *Z* < 1). Third, a χ^2^ test between the two periods for the number of participants for whom σ′ = 0 and the people for whom σ′ > 0 failed to show any Period effect for either both targets (*N* = 1 vs. 3; χ^2^ < 1) or left target (*N* = 5 vs. 3; χ^2^ < 1) or right target (*N* = 3 vs. 2; χ^2^ < 1). Disregarding the zeros, two further statistics were performed. Fourth, Wilcoxon test between the two periods failed to reach significance for either both or left or right targets (*Z* < 1). Fifth, KS test on cumulative histograms of the values across the whole population did not show any difference between the two periods for both targets (*D* = 0.333, *P* > 0.10), left target (*D* = 0.167, *P* > 0.10), and right target (*D* = 0.083, *P* > 0.10) (see Figure 2C).

#### Swivel vs. shift of distributions

For the group of participants, recinormal distributions differed between pre- and post-test periods for both targets (KS = 0.063, *P* = 0.001) and right target (KS = 0.080, *P* = 0.004), but not for left target (KS = 0.058, *P* = 0.086). The swivel vs. shift test favored a swivel of the distribution for targets either pooled or taken alone (*P* < 0.01) (see Table 5 and Figure 2C).

At the individual level, distributions of both targets differed between pre- and post-test periods in eight participants (KS, *P* < 0.05 in participants n°1–3, 5, and 10–12). The change took the form of a swivel in six participants (*P* < 0.01), in the sense that latency was reduced in five of them (n°1, 7, 10–12; see Table 5 and Figure 3).

For left target, distributions differed between pre- and post-test periods in five participants (KS, *P* < 0.05 in participants n°1, 2, 5, 9, and 12). There was a significant swivel of the distribution in seven participants (*P* < 0.01), in that latency was reduced in five of them (n°1, 7, 10–12; see Table 5 and Figure 4).

Finally for right target, distributions were different between the two periods in four participants (KS, *P* < 0.05 in participants n°1, 2, and 9, 10). The distribution significantly swivelled in eight participants (*P* < 0.01), and the latency decreased in six of them (n°1, 3, 7, and 10–12; see Table 5 and Figure 5).

### Trainer effects

Two instructors separately trained participants of two distinct groups. There was no difference in the results between the two groups, as is developed in the Appendix (see Trainer Effects).

### Additional analysis

As medication in participants n°9–11 may have led to a statistical bias, analyses were re-run by removing them from the group. Results were as in the main analysis, as is developed in the Appendix (see Additional Analysis).

### Control experiment

To examine test-retest effect, another six elderly fallers underwent two saccadic recordings separated by 10 weeks without any training. We failed to show any change between test and retest as developed in the Appendix (see Control Experiment).

## Discussion

The present study examined the effects of a motor activity, namely FP, on the motor control of elderly fallers as assessed by ocular saccade timing and dynamics. The main findings were as follows. First, FP reduced the rate of anticipatory saccades (<80 ms) in both valid and invalid directions, that is for, respectively, correct and erratic responses. Second, FP tended to enhance the rate of express saccades (80–134 ms) in valid direction, particularly to the left side, resulting in higher left-right symmetry of express triggering, while it tended to reduce the rate of express saccades in invalid direction. Third, FP reduced the mean latency of saccades and tended to reduce the within-participant variability of latency. Fourth, FP reduced the variability of saccade duration, amplitude, and peak velocity. Fifth, LATER analysis revealed that FP increased μ and failed to influence σ or σ′. Finally, the influence of FP on motor control took the form of a swiveling of the recinormal distributions in LATER analysis, which was clearly visible in several participants. The following sections discuss methodological issues before addressing the core subjects of the effects of motor activity on timing and dynamics of saccades. The discussion ends by offering a model to account for early saccade release.

### Methodological issues

Intervention studies lead to much lower sample sizes than cross-sectional ones do, due the difficulty and cost of conducting follow-up studies and to possible dropping out during the intervention period. For those reasons, our study was conduced on 12 participants, all of them women, consistent with other studies which have been conducted with such a sample size (e.g., *N* = 13 in Alpert et al., 2009). The relatively wide age range (27 years) was also due to the difficulty in enrolling older adults, but was nevertheless lower or comparable to those of previous intervention studies: range = 22 years in Marmeleira et al. (2009); 36 years in Alpert et al. (2009); 44 years in Emery et al. (2003). Finally, three of our participants were under medication, which may have influenced some of the results. The additional analysis excluding these participants showed similar results to those of the main analysis, suggesting that paroxetine, lithium, and gabapentine did not interfere with saccadic behavior or motor training, as used to be the case for drugs such as benzodiazepines or hypnotics (Abel and Hertle, 1988).

In intervention studies, changes between pre- and post-test periods can be attributed to (1) the time that has elapsed between the two examination periods; (2) test-retest effects; (3) the trainer; (4) the training activity without specificity; and/or (5) the training activity with some specificity as compared to other forms of training. In this study, the control experiment without motor training failed to show any effect between pre- and post-test periods, suggesting that the effects obtained in the main experiment were not due to variables (1) and (2). The lack of trainer effect also ruled out our results being due to variable (3). Rather, we suggest that the effects in the main experiment originate from the motor training itself (4), though it may be premature to conclude about the specificity of FP compared to other types of motor training (e.g., martial arts, dance).

### Variability of saccade dynamics

The first issue that needs discussing is that FP reduced the within-participant variability of saccade duration, amplitude, and peak velocity. A previous cross-sectional study showed that older women (71.1 ± 5.5 years) who regularly practice a “physical activity” have a higher accuracy of saccades (as assessed by saccade amplitude divided by target amplitude, i.e., gain) than age-matched non-sporting women (Gauchard et al., 2003). Our intervention study did not influence saccade gain but the variability of saccade dynamics, which was not reported in Gauchard et al.'s study. Such a discrepancy for gain might be due to the difference in the content of training activities. Indeed physical activity stands for the training of cardiovascular condition and strength and refers to activities such as running, cycling, swimming, etc. (Kramer and Erickson, 2007; Hillman et al., 2008), whereas motor activity is defined as the motor learning of skills such as balance, motor coordination, motor flexibility, and motor speed and relates to activities such as FP, dance, martial arts, etc. (Voelcker-Rehage et al., 2010). Though most activities may have an impact on cardiovascular, strength, balance, coordination, flexibility, and/or speed components, there is neurobiological grounding to distinguish between physical-dominant vs. motor-dominant activities (see below). In Gauchard et al.'s study (2003), the “physical activity” practiced by sporting combined physical (jogging and swimming) and motor (soft gymnastics and yoga) activities, whereas our study mainly focused on motor activity.

Velocity and duration of saccades are linearly linked to their amplitude, at least up to 20° horizontally, according to the so-called “main sequence” (Bahill et al., 1975). Thus, it is logical that a change in variability of peak velocity and of duration was associated with a similar one in amplitude. To account for the decrease in variability of saccade duration and peak velocity after training, it is suggested that FP induced a change in the cerebellum. Indeed, the cerebellum is known to be the neural basis of motor learning in that it guides muscular periphery during the acquisition of new motor skills (Glickstein and Doron, 2008). In the field of eye movements, “saccadic adaptation” has been a useful tool to explore motor skill learning (Thier et al., 2002). In the seminal McLaughlin paradigm, the subject is asked to saccade from an initial fixation point to a peripheral target and the target is displaced to a different eccentricity following the initiation of the saccade. Since the eyes land at the original target location rather the actual one, the subject gradually learns over repetitions to update motor commands so as to correct the error by adjusting the amplitude of saccade toward the final target location (McLaughlin, 1967). Over forty years of behavioral and neurophysiological research have clarified the different types of adaptation over different time scales and the related changes in the simple vs. complex firing properties of Purkinje cells (Prsa and Thier, 2011). The oculomotor vermis (i.e., the posterior part of the central cerebellum) receives two inputs: one from climbing fibers originates in the medial accessory nucleus of the inferior olive; and another one from mossy fibers originates in nuclei in the brainstem, namely the medial vestibular nucleus, nucleus prepositus hypoglossi, nucleus reticularis tegmenti pontis, pontine nuclei, paramedian pontine reticular formation (PPRF) and pontine raphe. Purkinje cells integrate these inputs and in turn project to the caudal fastigial nucleus (cFN), which contacts several structures, particularly controlateral PPRF containing the excitatory burst neurons that elicit horizontal saccades (Thier et al., 2002; Prsa and Thier, 2011). A lesion (Goldberg et al., 1993) or inactivation (Robinson et al., 1993) of cFN results in saccade dysmetria—that is higher variability of saccade endpoints—suggesting that the cerebellum contains a mechanism that continuously calibrates the oculomotor system to ensure accurate movements. According to Prsa and Thier (2011), this cerebellar mechanism is based on the optimization of movement duration to counteract internal variability continuously in the motor commands and adapt saccade amplitude. Our study suggests that FP training boosted such a cerebellar mechanism, even in elderly people with a falling history, resulting in reduced variability of saccade duration, thus of peak velocity and of amplitude. Consistent with this cerebellar hypothesis, Black et al. (1990) trained female rats for 30 days to either an acrobatic program consisting of balance beams, see-saws, rope bridges and other obstacles on elevated paths (i.e., a motor activity equivalent to our FP training), or to forced or voluntary exercise consisting in walking on a treadmill or having free access to a running wheel (i.e., a physical activity equivalent to walking or running in humans). Interestingly, tissue analysis of their cerebellar paramedian lobules evidenced synatogenesis (a greater number of synapses per cell) after acrobatic training, whereas forced/voluntary exercise led to angiogenesis (a greater density of blood vessels). Our study concludes that the mechanism underlying reduced variability of saccade duration, velocity, and amplitude by FP may have acted in the cerebellum through synaptogenesis.

### Saccade latency decrease and early triggering

The second issue that needs discussing is that FP reduced saccade mean latency and significantly modified the triggering of anticipatory (<80 ms) and of express saccades (80–134 ms). Our mean latency decrease is consistent with a previous study showing that sporting older women have lower latency than age-matched non-sporting women (Gauchard et al., 2003). As FP increased the rate of slow regular saccades, the decrease in mean latency might have been caused by the increase in the rate of express saccades rather than a general decrease in the main distribution of latencies. Indeed, FP tended to enhance the rate of express saccades in valid direction, while it tended to simultaneously reduce that of express saccades in invalid direction. Together with the fact that FP reduced the rate of anticipatory saccades in both valid and invalid directions, this suggests that FP improved saccadic control by vanishing anticipatory motor responses and boosting reflexive motor behavior. This hypothesis is in line with a previous study suggesting that older fallers exhibit fewer short-latency saccades than age-matched non-fallers (Yang et al., 2008). Though errors were not analyzed and the number of trials per condition was 10, making unlikely any analysis of latency distribution, this study points to a deficit in express triggering in fallers (Yang et al., 2008). Our study indicates that FP might be able to rehabilitate reflexive behavior in older fallers, making them more prone to react to unpredictable events thus avoiding new falls.

Left-right asymmetry was observed in the triggering of express saccades. Indeed before training, the rate of leftward express saccades was lower than that of rightward ones. This observation is consistent with previous studies showing a rightward bias in perception (e.g., Coubard et al., 2011) or action (e.g., Coubard and Kapoula, 2008). Specifically for eye movements toward basic stimuli (as in the present study), a rightward bias was reported for latency of visually guided saccades (Pirozzolo and Rayner, 1980; Hutton and Palet, 1986), for express saccades (Weber and Fischer, 1995; Honda, 2002), and for microsaccades during fixation (Hafed and Clark, 2002; Abadi and Gowen, 2004) or symmetrical vergence (Coubard and Kapoula, 2008). This bias can be interpreted as the result of hemispheric asymmetry in right-handed participants (Heilman et al., 1993; Kinsbourne, 1993) and/or of left-right reading direction of Latin-based language speakers (Singh et al., 2000). Interestingly, the present study showed that FP was able to boost express triggering to the left side, resulting in higher left-right symmetry in eliciting short-latency eye movements, which might favor daily life reactivity bilaterally in older adults.

Since their discovery in humans (Fischer and Ramsperger, 1984), express saccades have been subjected to extensive investigation to advance our understanding of the circumstances of their occurrence (Findlay and Walker, 1999) and of their underlying neural basis (Schiller and Tehovnik, 2005). There is some consensus today that express saccades rely on a subcortical retino-collicular route (Isa and Hall, 2009), while regular saccades results from attentional/decisional mechanisms involving a wide cortical network (Isa and Kobayashi, 2004). How the switching between cortical and subcortical pathways occurs, and how FP may favor such switching thus boosting reflexive behavior can be illuminated by LATER analysis. Consistent with the decrease in mean latency, FP significantly enhanced μ though it failed to influence neither σ nor σ′. More interesting was the observation that the effect of FP on motor control took the form of a swiveling of the recinormal distributions, which was visible in several participants. In LATER terms (see Introduction), the swivel indicates that a change has occurred in the distance between decisional thresholds. Our study concludes that initial threshold *S*_0_ has been elevated and/or final threshold *S*_*T*_ has been lowered by FP.

### The timer-rider model

**Figure 6:**
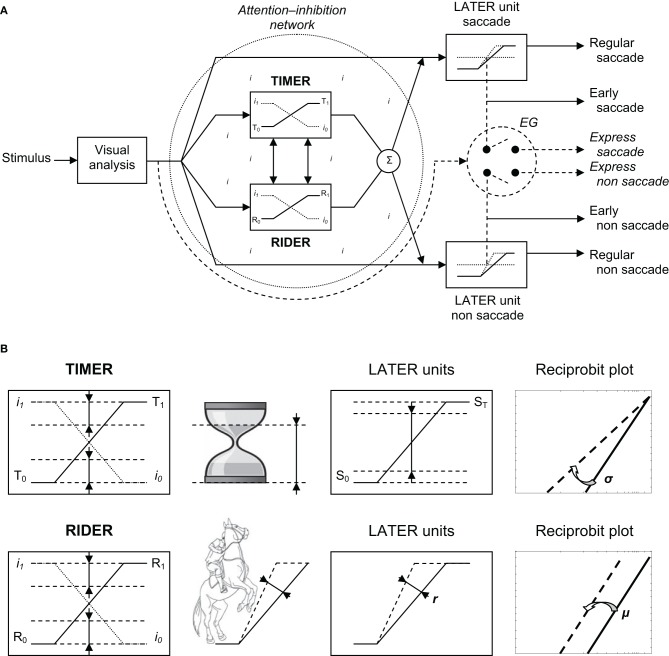
**(A)** TIMER-RIDER model. Visual information from the two retinas is analyzed to determine the location of the stimulus in space. Movement activation (right): For the decision to be made to elicit an eye movement toward the visual stimulus, decision signals initiate in LATER units for saccade and non-saccade, which rise at a constant rate to reach the threshold at which regular or early saccade and/or non-saccade are triggered. In LATER units, the thresholds and/or the rates of rise for saccade and non-saccade can differ. Movement inhibition (dotted circle): In a global attention-inhibition network, the inhibition process has value between *I*_1_ and *I*_0_ throughout the network. Two units modulate movement inhibition: TIMER (Threshold Interval Modulation with Early Release) and RIDER (Rate of rIse Deviation with Early Release). TIMER and RIDER signals increase from, respectively, values *T*_0_ and *R*_0_ to values *T*_1_ and *R*_1_ (full line), causing a mirror decrease in inhibition from *I*_1_ to *I*_0_ (dotted line). Modulators' effects are added (Σ) producing a change in LATER units in either decision thresholds or in decision gain or in both. Early and express triggering (right): Under strong conditions of attention-inhibition release by TIMER-RIDER modulators, LATER units produce short-latency eye movements in the form of early saccades. Under full conditions of attention-inhibition release, the short visual route bypassing attentional/decisional mechanisms (dashed arrow) elicit express saccades in an all or nothing way (switch). For non-saccade, there exist some uncertainties on the express nature of the movement and on whether express non-saccade is triggered by the same structure. **(B)** Effects of TIMER-RIDER on the inhibition process and on LATER units. An increase in the TIMER signal (T) causes a mirror reduction in the inhibition process I, and a reduction in the distance between initial threshold *S*_0_ and final threshold *S*_*T*_ of LATER units, resulting in a swivel of the recinormal distribution. An increase in the RIDER signal (R) also causes a mirror reduction in the inhibition process I, and an enhancement of the rate of rise *r* in LATER units, resulting in a shift of the recinormal distribution.

To account for the results, we would like to introduce a new model—the Threshold Interval Modulation with Early Release-Rate of rIse Deviation with Early Release (TIMER-RIDER) model—based on the LATER model (Carpenter, 1999) and on a model of saccade and vergence triggering (Coubard, 2011, pp. 195–196). The model is illustrated in Figure 6. In this model, excitatory vs. inhibitory mechanisms are distinguished for eliciting vs. suppressing movement. The former are embodied in LATER units, the latter takes the form of a global network lodging two *modulators* for down-regulating the inhibition process: TIMER and RIDER.

Visual information from the retina of each eye is processed to build a percept of stimulus location in space. In terms of excitatory mechanisms, a LATER unit is dedicated to saccade to respond to a stimulus step in direction (Figure 6A, Right/up). In this unit, the decision signal to elicit a saccade starts at an initial level *S*_0_ and rises at a constant rate *r* until it reaches a threshold value *S*_*T*_ at which point a regular saccade is triggered (Carpenter, 1999). Other LATER units are dedicated to non-saccade movement (i.e., pursuit, step or smooth vergence, etc.) to respond to some change of stimulus location other than step in direction (respectively, smooth in direction, step or smooth in depth, etc.) (Figure 6A, Right/down). To respond to a combined change of stimulus location, decision to generate saccadic and non-saccadic movements in LATER units is made at the same time, though their thresholds and/or rates of rise can differ (Takagi et al., 1995). In our study, stimuli were projected on a fronto-parallel plane thus saccades were not pure but combined with a slight change in depth, suggesting that a LATER unit for vergence might have been recruited though at a lower extent compared to that for saccade (Coubard, 2011).

In terms of inhibitory mechanisms, a network is devoted to attention-inhibition control (Figure 6A, Left). Such a network is hypothesized to be global rather than acting through local inhibitory modules onto local excitatory units (Coubard, 2011). The inhibition process has a value between *I*_1_ (maximum) and *I*_0_ (minimum) throughout the network, thus defining a certain “attentional state” which fluctuates over time. The attention-inhibition network delays the start of decision to generate a movement in LATER units allowing required parallel cognitive processes to result in the most appropriate decision. As a novelty, we would like to suggest the existence of two modulators within the attention-inhibition network. The first modulator is called TIMER, which stands for Threshold Interval Modulation with Early Release. TIMER enables LATER units to reduce the distance between their initial and final thresholds *S*_0_ and *S*_*T*_, respectively. TIMER also recalls an hourglass with decreasing distance between the level of sand in its upper part and the baseline of its support (see Figure 6B). The second modulator is called RIDER, for Rate of rIse Deviation with Early Release. RIDER allows LATER units to enhance their rates of rise *r*. RIDER is reminiscent of a horseman spiriting his mount to enhance the slope of the rearing up (see Figure 6B). As so-called modulators and contrary to LATER units, the activation of TIMER-RIDER does not elicit movement and conversely their inactivation does not prevent movement. We suggest that TIMER and RIDER signals (the *T* and *R* signals) rise independently, though the two modulators can interact, causing a mirror decrease in the process of inhibition (the *I* signal). Indeed when activated, TIMER and RIDER signals start at a minimal value—*T*_0_ and *R*_0_—corresponding to the highest level of inhibition *I*_1_ at which point movement is completely suppressed, then linearly rise until a maximal value—*T*_1_ and *R*_1_—corresponding to the lowest level of inhibition *I*_0_ at which movement is completely released. TIMER and RIDER activities are added thus producing in LATER units either a change in decisional thresholds or in the rates of rise or a combination of both.

Whenever internal or external contingencies call for short-latency responses, we suggest that two types of responses may occur. First under optimal conditions of “attention-inhibition release,” the reduction in the distance between initial and final thresholds and/or the enhancement of their rates of rise in LATER units may be extreme, thus enabling the triggering of early saccade or non-saccade (see Figure 6A). We speculate that it is the function of both TIMER and RIDER through their modulation of LATER units to make such an optimal release of attention-inhibition happen. Second under complete turn off of attention-inhibition, the entire attention-inhibition network and related decisional mechanisms may be bypassed enabling a shorter visual route and its express generator to elicit an express eye movement (see Figure 6A). In humans, such express eye movements have been evidenced for saccade (Fischer and Ramsperger, 1984), for convergence (Coubard and Kapoula, 2006), but not for pursuit (Kimmig et al., 2002). Such express triggering occurs in an “all or nothing” manner that would explain both the bimodality of latency distribution and the distinct metrics of express vs. regular saccades (Findlay and Walker, 1999). To summarize, the first type would be a very fast motor response which is nevertheless still under control, whereas the second type would be an uncontrolled motor response due to attentional/decisional failure. Future investigation will have to define the mathematical functions governing on the one hand the rise of TIMER signal and the threshold interval decrease in LATER units, and on the other hand the rise of RIDER signal and that of LATER units (see Figure 6B).

### Neural hypotheses of the timer-rider model and of fall prevention effects

How such a model may be implemented in the brain and by which mechanism FP training may have influenced either TIMER and/or RIDER in fallers is the final issue we now discuss.

In terms of excitatory mechanisms, oculomotor muscles are innervated by motor neurons which in turn are innervated by burst neurons in the brainstem. Different groups of burst neurons discharge for different types of eye movements. For horizontal saccades, burst neurons are located in the PPRF. Burst neurons generate two forces: a pulse that rotates the eyes and a step that maintains the eyes in eccentricity against the elastic forces of the oculomotor plant (reviewed by Scudder et al., 2002). At a higher level, excitatory neurons have been identified as showing a progressive increase in their firing rate prior to the activity of burst neurons: long-lead burst neurons in the brainstem (LLBN) (Kaneko, 2006), movement neurons in the superior colliculus (SC) (Dorris and Munoz, 1998), in the caudate nucleus (Lauwereyns et al., 2002) and in the frontal eye field (FEF) (Hanes and Schall, 1996). LATER units may be embodied in such LLBNs in the brainstem and movement neurons in the SC, caudate nucleus and FEF. At the lowest level, motor neurons and burst neurons are supposed not to participate to the LATER activity as their modulation occurs during the ballistic period of movement production (Boucher et al., 2007).

In terms of inhibitory mechanisms, a network involving brain areas from the brainstem to prefrontal cortex is devoted to attention-inhibition control of movement. At the lowest level, omnipause neurons (OPN) located in the pontine raphe exert monosynaptic inhibition on burst neurons. OPN activity is common to different groups of burst neurons (Scudder et al., 2002). At a higher level of the inhibitory hierarchy, fixation neurons have been evidenced in the SC (Munoz and Wurtz, 1993), the substancia nigra (Hikosaka and Wurtz, 1983), the FEF (Segraves and Goldberg, 1987) and the dorsolateral prefrontal cortex (DLPFC) (Tinsley and Everling, 2002). The activity of such fixation neurons may correspond to that of *I* signal described above. Indeed in mirror to movement neurons, fixation neurons progressively decreased their firing rate prior to saccade initiation. As for motor neurons and burst neurons, OPNs have a different status from fixation neurons since OPNs are modulated only during the ballistic period of movement during which movement cannot be suppressed (Boucher et al., 2007). Thus, OPNs do not belong to the attention-inhibition network as is presented here. The attention-inhibition network is hypothesized to be global rather than acting through local modules since modules of fixation neurons do not always respond to local units of movement neurons (e.g., in the substancia nigra).

Parallel to the long sensorimotor route of decisional and of attention-inhibition mechanisms, the shorter route enabling express triggering is embodied in the retino-collicular pathway. In response to a visual stimulus, monkey visual neurons of the superficial layers of SC are activated in ~40 ms bypassing the attention-inhibition network. In turn motor neurons of the deep layers of SC can produce a saccade in ~20 ms (Sparks, 1975). Under bypass of decisional and attentional mechanisms (see above), a switch enables visual neurons to directly activate motor neurons in SC, probably through the interlaminar connection between its superficial and deep layers (Isa and Kobayashi, 2004). The SC is critical for the express triggering of saccades (reviewed by Schiller and Tehovnik, 2005) and may play a similar role for vergence (Chaturvedi and van Gisbergen, 2000), but there exits uncertainties about other types of eye movements. Recent advances in the LATER model have suggested some possibility of distinguishing between express vs. early saccades even in a reciprobit plot (Noorani and Carpenter, 2011). Specifically, express saccades having a distinct peak in a frequency histogram might result in a population lying parallel to the main distribution, while early saccades lying on the line with intercept of 0.5 may correspond to short-latency saccades without a distinct peak in a frequency histogram (Noorani and Carpenter, 2011) (see Figures 1B,C). In line with this proposal, it is suggested that between controlled regular and uncontrolled express eye movements, “early eye movements” may represent an intermediate situation in which controlled eye movements are triggered not by the express generator but LATER units having extremely decreased distance between their initial and final thresholds and/or enhanced rates of rise.

How the two hypothesized modulators may be implemented in the brain has been enlightened by recent brain imaging findings in humans. Indeed, Domenech and Dreher (2010) examined the neural correlate of the LATER model. Using fMRI in 14 healthy young adults, they showed differential activations in the anterior cingulate cortex (ACC) and the right dorsolateral prefrontal cortex (rDLPFC). Specifically, ACC's BOLD activity was positively correlated to the distance to the decisional threshold but not to the slope of the accumulation of sensory evidence. In contrast, rDLPFC's BOLD activity was positively correlated to the slope of sensory evidence accumulation but not to the distance to the decisional threshold. With regard to our model, the TIMER signal may correspond as first approximation to ACC activity, while the RIDER signal is a better fit for rDLPFC activity. Our result showed that FP training produced a reciprobit swivel in which latency was reduced. In terms of the TIMER-RIDER model, FP might have boosted the TIMER modulator thus allowing the promotion of early saccade triggering. Related to the neural correlate, this means that FP training might have favored the activity of ACC thus enabling decision threshold modulation rather than decision gain control (Domenech and Dreher, 2010).

To explain the effect of FP on threshold modulation in elderly fallers, we may posit that the practice of motor activity may have stimulated cortical-subcortical circuitry. Motor control involves extensive areas of the central nervous system from the spinal cord to the cerebral cortex, such as globus pallidus, putamen, caudate nucleus, thalamus, substancia nigra, subthalamic nucleus, cerebellum, reticular formation, vestibular nuclei. Motor activity practiced in FP may have stimulated cortical-subcortical loops, specifically the anterior cingulate loop linking ACC to striatum, pallidum, substancia nigra and thalamus (Alexander et al., 1986). Consistent with this assumption, an fMRI study showed the involvement of ACC—together with that of DLPFC, superior frontal and parietal cortex, inferior parietal and temporal cortex—in high-fit (either motor or physical) adults aged 62–79 years performing a perceptual speed task, as compared to low-fit (either motor or physical) age-matched participants (Voelcker-Rehage et al., 2010). Practice of motor activity such as FP may reinforce ACC and counteract its aging, consistent with its under-recruitment when high-motor fit older adults are compared to high-physically fit controls (Voelcker-Rehage et al., 2010).

## Conclusion

Due to the complexity of balance control and to the variety of deficits that can produce balance decline in aging, dynamic models of motor control provide a useful tool to help researchers understand how motor training improve central motor processing of balance in aging. Using such a Bayesian decision-making model in older fallers, this study has shown that a FP program promoted the triggering of express saccades by modulating decisional thresholds, which may be related to a change in cortical-subcortical activity involving ACC. FP also reduced within-participant variability of saccadic duration, amplitude and peak velocity, which may have been due to some modulation of the cerebellar circuitry. This study provides useful directions for future therapeutic interventions in elderly fallers.

### Conflict of interest statement

The author declares that the research was conducted in the absence of any commercial or financial relationships that could be construed as a potential conflict of interest.

## Appendix

### Trainer effects

Two instructors separately trained participants n°1, 3–6, 9, and 11–12 on the one hand, and participants n°2, 7, 8, and 10 on the other. ANOVAs with Trainer as between-participant factor (2 levels: instructor 1 or 2) and Target location (2 levels: left or right) and Period (2 levels: pre- or post-test period) as within-participant factors on 10 rates (rates of anticipatory, express, fast regular, slow regular, late, very late saccades in valid direction; rates of anticipatory, slow regular, late, and very late saccades in invalid direction; there were insufficient erratic express and fast regular saccades to perform statistics), on 9 measures of timing and dynamics (mean and variability of latency, duration, amplitude, and peak velocity; gain), and on 3 LATER measures (u, o, and o') showed neither Trainer effect nor interaction between Trainer and other factors (*P* > 0.05).

### Additional analysis

We re-ran analyses for saccade timing and dynamics (mean latency; variability of latency, of duration, of amplitude, and of peak velocity) by removing from the group participants n°9–11 who were under medication.

For mean latency, ANOVA with Target location and Period as factors showed a main effect of Period [*F*_(1, 8)_ = 5.87, *P* = 0.042] with decreasing latency between pre- (298.8 ms) and post-test (273.3 ms) periods. For variability of latency, the Period effect was significant [*F*_(1, 8)_ = 5.53, *P* = 0.047] with a tendency for decreasing values between pre- (116.9 ms) and post-test (98.6 ms) periods.

There was a main effect of Period for variability of duration [*F*_(1, 8)_ = 9.20, *P* = 0.016], variability of amplitude [*F*_(1, 8)_ = 10.3, *P* = 0.012], and variability of peak velocity [*F*_(1, 8)_ = 8.30, *P* = 0.020] with decreasing values between the two periods for all parameters (9.6 vs. 6.5 ms, 2.1 vs. 1.4°, 65.4 vs. 43.5°/s for, respectively, duration, amplitude and peak velocity).

### Control experiment

#### Methods

Six other French women, aged 79.4 (Mean) ± 1.7 (SD) years (range = 77.9–81.4 years), gave their informed consent to participate in the study. They were right-handed, had normal or corrected-to-normal vision, and were unaware of the goal of the experiment. They had 10.0 ± 2.8 years of education, and scored 3.0 ± 0.7 to the 1–4 socio-cultural scale (Kalafat et al., 2003). They scored 28.2 ± 1.3 to the MMSE (Folstein et al., 1975), and 11.4 ± 2.3 to the WAIS code (Wechsler, 1989). They were recruited from a waiting list of older adults with falling history, who had been given a medical prescription for fall prevention training. None of them were under medication. They underwent two saccadic recordings, separated by 10 weeks during which they did not perform any training. Procedure and analyses were as in the main experiment.

### Results

Results are detailed in Tables A1,2 for, respectively latency, duration, amplitude, and peak velocity for every participant and for the group. We were only interested in the effect of Period, not Direction, so we performed a Student's *t* test for timing and dynamics between the two periods.

**Table A1:** **Mean ± Standard deviation of latency and of duration in milliseconds for both targets in the two periods, for every participant and the group of participants (All)**.

	**Pre**	**Post**	**Pre**	**Post**
P1	275.3 ± 36.2	281.6 ± 114.6	52.3 ± 3.7	51.7 ± 7.2
P2	310.5 ± 94.9	360.6 ± 165.6	50.3 ± 4.1	52.1 ± 7.7
P3	282.7 ± 116.4	270.7 ± 120.4	51.8 ± 6.3	52.7 ± 11.3
P4	372.0 ± 209.7	326.8 ± 119.0	49.0 ± 8.8	50.2 ± 7.0
P5	299.6 ± 94.2	318.4 ± 83.3	50.0 ± 4.7	53.9 ± 6.2
P6	298.7 ± 159.6	285.7 ± 60.3	52.6 ± 16.0	54.2 ± 6.5
All	306.5 ± 118.5	307.3 ± 110.5	51.0 ± 7.3	52.5 ± 7.7

**Table A2:** **Mean ± Standard deviation of amplitude in degrees and of peak velocity in degrees per second for both targets in the two periods, for every participant and the group of participants (All)**.

	**Pre**	**Post**	**Pre**	**Post**
P1	9.9 ± 0.7	9.5 ± 2.0	321.4 ± 26.3	323.2 ± 76.0
P2	9.7 ± 1.4	10.0 ± 2.0	340.4 ± 43.5	347.3 ± 57.7
P3	10.1 ± 1.5	9.8 ± 2.5	389.4 ± 59.5	364.7 ± 81.1
P4	10.5 ± 3.7	9.9 ± 1.8	383.4 ± 93.3	433.3 ± 62.6
P5	8.2 ± 0.8	9.7 ± 1.9	307.3 ± 40.3	303.8 ± 57.0
P6	11.4 ± 4.9	9.9 ± 1.6	435.4 ± 156.8	343.1 ± 48.3
All	9.9 ± 2.2	9.8 ± 2.0	362.9 ± 70.0	352.6 ± 63.8

There was no effect of Period for mean latency (*t*_5_ < 1) nor for variability of latency (*t*_5_ < 1). There was no effect of Period on duration (*t*_5_ = −2.45, *P* = 0.058), amplitude (*t*_5_ < 1), and peak velocity (*t*_5_ < 1), nor on variability of duration (*t*_5_ < 1), of amplitude (*t*_5_ < 1), and of peak velocity (*t*_5_ < 1).
